# Health-Related Quality of Life of Adolescents With Non-transfusion-Dependent Thalassemia in Basrah, Iraq

**DOI:** 10.7759/cureus.70189

**Published:** 2024-09-25

**Authors:** Zahraa M Hameed, Meaad K Hassan, Bahaa A Ahmed

**Affiliations:** 1 Department of Pediatrics, Basrah Teaching Hospital, Basrah, IRQ; 2 Department of Pediatrics, College of Medicine, University of Basrah, Basrah, IRQ

**Keywords:** alpha-thalassemia intermedia, beta-thalassemia minor, heath-related quality of life, non-transfusion-dependent thalassemia, thalassemia intermedia

## Abstract

Background: Thalassemia is a chronic inherited disease with the potential for serious clinical and psychological effects. In the case of thalassemia, a cure is not currently accessible, and lifelong treatment is required. Health-related quality of life (HRQoL) is considered a crucial health outcome.

Objectives: This study aims to assess the HRQoL of children and adolescents with non-transfusion-dependent thalassemia (NTDT) and compare it with that of beta-thalassemia major (β-TM) and healthy subjects.

Patients and methods: This case-control study included 88 patients with NTDT and 153 age- and gender-matched healthy children and adolescents. In addition, we included 70 registered patients with β-TM. We used the short-form health survey questionnaire to assess HRQoL.

Results: Of the 88 patients, 41 were diagnosed with alpha-thalassemia intermedia (α-TI; hemoglobin H disease), and 47 were with beta-thalassemia intermedia (β-TI). HRQoL domains were significantly higher in healthy children and adolescents compared to NTDT patients (P<0.001); the role emotion domain was the most affected in NTDT patients (51.92 ± 3.37), followed by general health (52.72 ± 3.05) and role physical (53.59 ± 3.13). α-TI patients had significantly higher HRQoL domains than patients with β-TI. The study also indicated that NTDT patients had significantly better QoL scores compared to β-TM patients (P<0.001) across all domains.

Conclusions: NTDT patients have a lower HRQoL compared to healthy controls. However, their HRQoL scores are significantly better than those of patients with β-TM. Among NTDT patients, those with α-TI have significantly better HRQoL scores compared to patients with β-TI.

## Introduction

Non-transfusion-dependent thalassemia (NTDT) is a group of thalassemia disorders that include patients who do not require regular blood transfusions for survival. The most common types are beta-thalassemia intermedia (β-TI), hemoglobin E/β-thalassemia, and alpha-thalassemia intermedia (α-TI; hemoglobin H (HbH) disease) [1].

Patients with NTDT experience numerous clinical complications despite their independence from frequent transfusions. Morbidities in NTDT stem from the interaction of various pathophysiological factors: ineffective erythropoiesis, iron overload (IOL), and hypercoagulability [2].

The evaluation of quality of life (QoL) differs from other medical assessments as it centers on individuals’ perspectives of their health and appraises additional aspects of life, thereby providing a more comprehensive view of well-being [3].

Patients with NTDT may develop significant complications related to their disease, primarily IOL, and challenges in adhering to iron chelation therapy (ICT), which negatively affects their QoL [4]. Moreover, a significant percentage of patients with NTDT encounter severe cardiovascular, endocrine, hepatic, or skeletal complications [5].

Patients with NTDT present at the end of the clinical spectrum between the ages of two and six years but typically come to medical attention and are diagnosed in late childhood or during adolescence. This period can be a challenging time for them to adjust to their disease, as adolescence itself is a difficult phase. The presence of an illness may further lower their chance for psychosocial adaptation to the disease, decrease their understanding of it, or result in fewer interactions with comprehensive care centers and staff. These factors may harm their QoL [6,7].

The term “health-related quality of life (HRQoL)” is extensively employed in assessing the QoL in general and specifically in patients with chronic diseases. The 36-Item Short Form Survey version 2 (SF-36 v2) questionnaire is recommended as the standard instrument for assessing HRQoL in thalassemia, as the majority of the questionnaire’s subscales appear to be affected by complications from the disease and ICT [8].

This study evaluated the HRQoL in children and adolescents with NTDT. The objective was to compare it with those who are healthy and those with beta-thalassemia major (β-TM) within the same age group.

## Materials and methods

This case-control study included patients with NTDT registered at the Centre for Hereditary Blood Diseases (CHBD) in Basrah. A total of 88 patients with NTDT, aged 12-18 years, were recruited over seven months, from December 2017 through June 2018. Those who were deaf-mute and who refused to do or complete the interview were excluded from the study. The Scientific and Ethical Committee of the College of Medicine, University of Basrah approved the study (approval number: 7/35/716, date: February 13, 2020).

The data included socio-demographic variables as well as disease-related variables such as age at diagnosis, age at the first transfusion (if applicable), the number of clinic visits, hospital admissions, blood transfusions in the previous year, and a history of splenectomy. The patients and their caregivers were interviewed, and medical records were reviewed for disease-related complications (e.g., diabetes mellitus, bone disease, heart failure, growth retardation, delayed puberty, gall bladder diseases, and stroke), the use of ICT (type and duration), and the use of hydroxyurea. The family income was categorized based on the average monthly per capita in Iraqi Dinars (ID) as follows: low (<100,000 ID/month), medium (100,000-250,000 ID/month), and high (>250,000 ID/month) [9].

A total of 88 patients (34 males and 54 females) with NTDT were enrolled in this study. All of them were identified as having either β-TI or α-TI (HbH disease) based on clinical and laboratory findings [1,10]. Forty-seven patients were categorized with β-TI, and 41 patients presented with HbH disease.

The NTDT patients were categorized based on their status of blood transfusion as follows: never transfused, occasional blood transfusion (patients who require incidental blood transfusion due to transient severe anemia), and regular blood transfusion (once every one to three months) [11].

In addition to NTDT patients, 70 patients with β-TM of comparable age and gender who were registered at CHBD and consulted the center for routine follow-up and/or blood transfusion were also recruited during the study period. Verbal consent was obtained from children and adolescents, and/or their caregivers, for the completion of the data and the SF-36 v2 questionnaire.

For the NTDT and β-TM patients, data was collected through direct interviews with the NTDT patients and one of their parents who had consulted the CHBD for routine follow-ups and/or blood transfusions. Blood transfusion requirements vary between different groups, namely NTDT and β-TM, and are categorized as follows: none, occasional (1-5 times/year), low (6-12 times/year), and high (13-22 times/year) [12].

The control group comprised 153 age- and gender-matched children and adolescents with no history of any chronic conditions, including hemoglobinopathies, or health-related conditions four weeks before the submission of required responses for the study [13]. Data were collected from the control group through visits to six primary and secondary schools in various districts of Basrah. Before their recruitment into the study, informed verbal consent was obtained from the children, school managers, and teachers.

HRQoL (SF-36 v2) questionnaire

This type of questionnaire, a well-recognized, generic short-form health survey with 36 questions, can be self-administered or given by a trained interviewer. This survey measures the functional status, well-being, and overall evaluation of health from the patient’s perspective, with SF-36 v2 items acting as multiple operational indicators of health, including behavioral function and dysfunction, distress, and well-being [14]. These SF-36 v2 items are used to score eight dimensions, specifically physical functioning (PF), role physical (RF), bodily pain (BP), general health (GH), vitality (VT), social functioning (SF), role emotion (RE), and mental health (MH). These dimensions form the physical component summary and the mental component summary [13].

The median reliability coefficients for each of the eight scales are ≥0.08, except for SF, which has a median reliability across studies of 0.76. The questionnaire’s validity rests between 80% and 90%. An evaluation of the cross-cultural adaptations of this instrument indicated moderate to good quality [13].

The Arabic version of SF-36 v2 was utilized. The median Cronbach’s alpha for the Arabic RAND-36 exceeded 0.07 in most scales across multiple subgroups [15,16]. The scoring of this questionnaire translates an individual’s responses to a standard 0-100 scale. In the SF-36 v2 questionnaire, higher scores signify a better QoL, while lower scores indicate poor health and QoL.

Statistical analysis

Data was processed and analyzed using SPSS Statistics version 23 (IBM Corp. Released 2015. IBM SPSS Statistics for Windows, Version 23.0. Armonk, NY: IBM Corp.). Comparisons of proportions were carried out using the Chi-square test for cross-tabulation, provided each cell had an expected frequency of 5 or more. Fisher’s exact test was applied when one or more cells had an expected frequency of less than 5.

The independent sample t-test was used for the quantitative comparison between the means of two different samples. ANOVA was employed to assess the significance of the differences between the means of three samples. Multiple linear regression analysis was employed to evaluate the correlation between the SF-36 v2 scores of NTDT patients and the various studied socio-demographic and clinical variables, as well as disease-related complications. A P-value of <0.05 was considered statistically significant for all statistical tests.

## Results

Socio-demographic characteristics of patients with NTDT and healthy controls

A total of 153 healthy children and adolescents were evaluated. The mean age for NTDT patients was 14.07 ± 1.95, while the mean age for the healthy control group was 14.46 ± 2.05. NTDT patients exhibited a significantly lower educational level, lower parental education, and lower family income (P<0.001) (Table 1).

**Table 1:** Selected socio-demographic variables of NTDT patients and control group * Fisher’s exact test was used to assess the P-value, ** Chi-square test was used to assess the P-value NTDT: non-transfusion-dependent thalassemia, ID: Iraqi Dinars

Variables		NTDT patients total (88) N. (%)	Healthy children total (153) N. (%)	P-value
Gender	Males	34 (38.64)	62 (40.52)	0.773**
	Females	54 (61.36)	91 (59.48)	
Age (years)	12-15	51 (57.95)	87 (56.86)	0.869**
	16-18	37 (42.05)	66 (43.14)	
Residence	Center	32 (36.36)	117 (76.47)	0.000**
	Periphery	56 (63.64)	36 (23.53)	
Educational level of subject	Didn't attend	8 (9.09)	0 (0.00)	0.000*
	Primary	44 (50.00)	62 (40.52)	
	Secondary	36 (40.91)	91 (59.48)	
Educational level of father	Illiterate	7 (7.95)	2 (1.31)	0.000*
	Primary	44 (50.00)	24 (15.69)	
	Secondary	26 (29.55)	72 (47.05)	
	Higher	11 (12.50)	55 (35.95)	
Educational level of mother	Illiterate	18 (20.45)	2 (1.31)	0.000*
	Primary	41 (46.59)	42 (27.45)	
	Secondary	25 (28.41)	82 (53.59)	
	Higher	4 (4.55)	27 (17.65)	
Per capita income (ID/month)	Low	56 (63.64)	43 (28.10)	0.000*
	Medium	31 (35.23)	82 (53.60)	
	High	1 (1.13)	28 (18.30)	

Characteristics of NTDT patients

Of the 88 patients with NTDT, 46.59% had α-TI (HbH disease), and 53.41% had β-TI. The study did not reveal a significant difference between α-TI and β-TI in terms of age, gender, maternal educational level, and family income (P>0.05) (Table 2). The educational levels of patients and their fathers significantly differed concerning disease type; specifically, the percentage of those who did not attend school was significantly higher, and those who attended secondary school were lower in β-TI compared to HbH disease patients. We found a significantly higher number of β-TI patients who were on ICT and HU and required more blood than those with α-TI (P<0.001). Furthermore, the frequency of complications was significantly higher among β-TI patients (P<0.05). Among patients with HbH disease, 82.93% experienced no complications. This is in contrast to the 53.20% of β-TI patients who also had no complications. The most common complications among β-TI patients were cardiac complications, observed in eight patients (17.02%), followed by growth retardation in four patients (8.51%). Stroke and osteoporosis complications were each observed in three patients (6.38%). In patients with α-TI, the most common complications included osteoporosis in three (7.31%) cases, followed by all immunization and cardiac disease, with two (4.87%) cases each.

**Table 2:** Selected socio-demographic and clinical variables of patients with α-TI and β-TI * Fisher’s exact test was used to assess the P-value, ** Chi-square test was used to assess the P-value α-TI: α-thalassemia intermedia, β-TI: β-thalassemia intermedia; ID: Iraqi Dinars, ICT: iron chelation therapy, HU: hydroxyurea

Variables		α-TI patients total (41) N. (%)	β-TI patients total (47) N. (%)	P-value
Gender	Males	16 (39.02)	18 (38.30)	0.944**
	Females	25 (60.98)	29 (61.70)	
Age (years)	12-15	23 (56.10)	28 (59.60)	0.742**
	16-18	18 (43.90)	19 (40.40)	
Residence	Center	20 (48.78)	12 (25.53)	0.024**
	Periphery	21 (51.22)	35 (74.47)	
Educational level of subject	Did not attend	1 (2.44)	7 (14.90)	0.027*
	Primary	19 (46.34)	25 (53.19)	
	Secondary	21 (51.22)	15 (31.91)	
Educational level of father	Illiterate	1 (2.44)	6 (12.76)	0.048*
	Primary	21 (51.22)	23 (48.94)	
	Secondary	10 (24.39)	16 (34.04)	
	Higher	9 (21.95)	2 (4.26)	
Educational level of mother	Illiterate	6 (14.63)	12 (25.53)	0.293*
	Primary	19 (46.34)	22 (46.81)	
	Secondary	15 (36.59)	10 (21.28)	
	Higher	1 (2.44)	3 (6.38)	
Per capita income ID/month	Low	22 (53.66)	34 (72.34)	0.145*
	Medium	19 (46.34)	12 (25.53)	
	High	0 (0.00)	1 (2.13)	
Age at diagnosis (years)	2-5	19(46.30)	32(68.10)	0.052**
	>5	22(53.70)	15(31.90)	
Blood transfusion/last year	None	36 (87.80)	17 (36.17)	<0.001*
	Occasional	2 (4.88)	0 (0.00)	
	Regular	3 (7.32)	30 (63.83)	
Hospitalization/last year	None	35 (85.36)	31 (65.96)	0.083*
	<3	3 (7.32)	9 (19.15)	
	≥ 3	3 (7.32)	7 (14.89)	
Complications	None	34 (82.93)	25 (53.19)	0.003**
	Present	7 (17.07)	22 (46.81)	
ICT	Yes	5(12.20)	29(61.70)	<0.001**
	No	36(87.80)	18(38.30)	
HU	Yes	0(0.00)	8(17.00)	0.006*
	No	41(100.00)	39(83.00)	

Socio-demographic and clinical characteristics of patients with NTDT and β-TM

The study also included 70 patients with β-TM. There was no significant difference in all the studied variables between both groups, except for the age groups. The number of NTDT patients aged 12-15 years old was significantly higher (P<0.05). However, there was no significant difference among males and females of different age groups (P>0.05) (Table 3). The study reported a statistically significant difference in the age of diagnosis and frequency of blood transfusion between patients with NTDT and β-TM (P<0.05). Concerning disease-related complications, patients with β-TM have a significantly higher frequency of complications compared to NTDT patients, mainly cardiac complications (32.46%) and growth retardation (15.71%).

**Table 3:** Selected socio-demographic characteristics of NTDT and β-TM patients * Chi-square was used to assess the P-value, ** Fisher’s exact test was used to assess the P-value NTDT: non-transfusion-dependent thalassemia, β-TM: beta-thalassemia major, ID: Iraqi Dinars, BT: blood transfusion, HCV Ab: hepatitis C virus antibodies

Variables		Type of thalassemia		P-value
		NTDT total (88) N. (%)	β-TM total (70) N. (%)	
Gender	Males	34 (38.64)	33 (47.14)	0.332*
	Females	54 (61.36)	37 (52.86)	
Age (years)	12-15	51 (57.95)	27 (38.57)	0.015*
	16-18	37 (42.05)	43 (61.43)	
Residence	Center	32(36.36)	21(30.00)	0.400*
	Periphery	56 (63.64)	49 (70.00)	
Educational level of the subject	Did not attend	8 (9.09)	14 (20.00)	0.116*
	Primary	44 (50.00)	34 (48.57)	
	Secondary	36 (40.91)	22 (31.43)	
Educational level of father	Illiterate	7 (7.95)	9 (12.86)	0.570*
	Primary	44 (50.00)	28 (40.00)	
	Secondary	26 (29.55)	23 (32.86)	
	Higher	11 (12.50)	10 (14.28)	
Educational level of mother	Illiterate	18 (20.45)	13 (18.57)	0.792**
	Primary	41 (46.59)	38 (54.28)	
	Secondary	25 (28.41)	16 (22.86)	
	Higher	4 (4.55)	3 (4.29 )	
Per capita income ID/month	Low	56 (63.64)	41 (58.57)	0.166**
	Medium	31 (35.23)	24 (34.29)	
	High	1 (1.13)	5 (7.14)	
Age at diagnosis	<2 years	0 (0.00)	70 (100.00)	<0.001**
	2-5 years	51 (58.00)	0 (0.00)	
	>5 years	37 (42.00)	0 (0.00)	
Age at 1^st^ BT	<2 years	10 (17.24)	69 (98.57)	<0.001**
	≥2 years	48 (82.76)	1 (1.43)	
BT/last year	None	50 (56.80)	0 (0.00)	<0.001**
	Occasional	10 (11.36)	0 (0.00)	
	Low	13 (14.80)	24 (34.30)	
	High	15 (17.04)	46 (65.70)	
HCV Ab seropositive	Yes	5 (5.68)	40 (57.14)	<0.001*
	No	83 (94.32)	30 (42.86)	
Hospitalization/last year	None	66 (75.00)	52 (74.29)	0.742**
	>3	12 (13.64)	12 (17.14)	
	≥3	10 (11.36)	6 (8.57)	
Complications	None	59 (67.05)	32 (45.71)	0.009**
	Yes	29 (32.95)	38 (54.29)	

HRQoL domains among different studied groups

Patients with NTDT have significantly lower scores in all HRQoL domains compared to the control group. However, when compared to β-TM, NTDT patients had high scores and scored higher in all HRQoL domains (P<0.001) (Table 4). Patients with HbH disease generally have a better HRQoL across all domains as compared to β-TI, with the exception being the SF domain. The study also demonstrated a significant negative correlation between the frequency of blood transfusion and all domains. Additionally, there was a significant negative correlation between patient age and the GH and MHCS domains. A significant positive association was found between per capita income and most of the SF-36 v2 scores, except for PF and VT, which were not impacted by family income (Table 5). It was also observed that BP did not have any association with any of the studied variables.

**Table 4:** SF-36 v2 scores of NTDT and β-TM patients and control group * ANOVA test was used to assess the P-value NTDT: non-transfusion-dependent thalassemia, β-TM: beta-thalassemia major, PHCS: physical health component score, MHCS: mental health component score, SF-36 v2: 36-Item Short Form Survey version 2

Domains	NTDT patients total (88) mean ± SD	β-TM patients total (70) mean ± SD	Controls total (153) mean ± SD	P-value*
Physical functioning	67.95 ± 28.12	45.07 ± 20.92	89.19 ± 14.92	<0.001
Role physical	53.59 ± 29.23	33.27 ± 21.12	86.91 ± 18.94	<0.001
Role emotion	51.92 ± 31.70	31.90 ± 22.91	88.31 ± 17.42	<0.001
General health	52.72 ± 28.67	29.07 ± 18.67	83.46 ± 17.80	<0.001
Bodily pain	69.60 ± 25.67	54.64 ± 24.74	89.61 ± 14.87	<0.001
Social functioning	71.16 ± 26.75	58.03 ± 26.41	85.79 ± 19.18	<0.001
Vitality	65.12 ± 21.62	51.87 ± 19.65	84.25 ± 18.05	<0.001
Mental health	64.97 ± 22.02	49.78 ± 20.49	84.93 ± 18.83	<0.001
PHCS	61.79 ± 20.60	42.78 ± 15.34	86.69 ± 13.41	<0.001
MHCS	62.68 ± 22.81	46.62 ± 19.75	86.33 ± 15.84	<0.001

**Table 5:** Linear regression analysis of determinants on SF-36 v2 scores among NTDT patients SF-36 v2: 36-Item Short Form Survey version 2, NTDT: non-transfusion-dependent thalassemia

Variables	β-coefficient	P-value	R2
Physical functioning			
Number of blood transfusions	-0.335	0.026	0.374
Role physical			
Per capita income	0.235	0.038	0.363
Number of blood transfusions	-0.303	0.045	
Role emotion			
Per capita income	0.278	0.013	0.380
Number of blood transfusions	-0.328	0.028	
Social functioning			
Per capita income	0.232	0.044	0.377
Number of blood transfusions	-0.324	0.036	
Vitality			
Number of blood transfusions	0.304	0.049	0.336
Mental health			
Per capita income	0.283	0.010	0.407
Number of blood transfusions	0.306	0.036	
General health			
Per capita income	0.214	0.043	0.443
Number of blood transfusions	-0.375	0.009	
Age	-0.215	0.025	
Physical health component score			
Per capita income	0.222	0.027	0.502
Number of blood transfusions	-0.388	0.004	
Mental health component score			
Per capita income	0.310	0.004	0.451
Number of blood transfusions	- 0.377	0.008	
Age	-0.208	0.029	

## Discussion

NTDT encompasses a variety of phenotypes. Unlike patients with β-TM, those with NTDT do not require regular blood transfusions to live. The most common forms are β-TI, HbE/β-thalassemia, and α-TI (HbH disease) [1]. The effects of NTDT and TDT, along with their associated complications on HRQoL, remain largely unknown. The degree to which health is impaired, as perceived by the patients, is a crucial piece of information that needs to be highlighted to provide appropriate therapy [7].

This study reveals that HRQoL in NTDT patients was lower than that in healthy children and adolescents across all SF-36 v2 domains. This result aligns with the findings reported by Safizadeh et al. in Kerman, Iran, demonstrating that NTDT patients have moderate scores compared to healthy Iranian individuals. However, it should be noted that the mean age of their patients was 22.95 ± 4.82 years [17]. Such differences in QoL are to be expected, given the unique physical conditions and disease-related issues that thalassemia patients face [18].

Patients with NTDT were observed to have better HRQoL than β-TM patients in all SF-36 v2 scores, with the lowest score reported for RE. Next was GH and then RP. Patients with β-TM also reported the lowest scores for GH, RE, and RP alike. Potential causes may include chronic anemia and/or IOL. Chronic anemia can lead to extramedullary hematopoiesis (EMH) and facial changes, which subsequently result in lower self-esteem and heightened fatigability. This makes them more inclined to shun social and physical activity [18]. The RE domain is affected, as patients with thalassemia often feel a sense of otherness when compared to their peers. These patients may develop negative beliefs about their lives, occasionally experiencing feelings of sadness, anger, and hurt toward their illness. Furthermore, the treatment they undergo, which includes blood transfusions, recurrent invasive procedures, and hospital visits, can have adverse effects on their emotional and physical well-being [19]. A prior study in Basra reported that 48.2% of TI children exhibit growth retardation and 11.8% show signs of EMH, both of which can affect self-esteem and, subsequently, physical activity [20].

The superior HRQoL scores for NTDT patients may be due to them experiencing fewer disease-related complications, infrequent blood transfusions, and less need for ICT than β-TM patients, particularly those with HbH disease [21]. These superior HRQoL scores for NTDT patients when compared with β-TM patients are consistent with findings by Mikael and Al-Allawi in Kurdistan, Iraq [22]. However, these results are at odds with a study by Musallam et al. in Lebanon that assessed the HRQoL of 32 β-TI and 48 β-TM patients using the SF-36 v2. They found adult patients with β-TI had lower HRQoL than β-TM patients. They reasoned that β-TM patients had a longer time, thus a greater opportunity to adapt to the disease, its complications, and treatments. They also posited that despite both thalassemia types being managed at the same center, the β-TM patients, more frequently present, would become more familiar with the medical staff, form stronger bonds with each other and the healthcare staff, and consequently acquire more knowledge about the disease, its complications, and better adapt to the nature of the disease [23]. A different study by Cappellini et al. noted that NTDT patients had significantly lower scores in GH, vitality, and mental component scores, without significant differences in other domains [24].

Additionally, the healthcare system has been giving significant attention to β-TM, even though this focus has gradually started shifting towards β-TI and its severity [25]. Patients with β-TI tend to have lower HRQoL scores in all domains compared to those with HbH disease. This discrepancy can be largely attributed to the higher frequency of complications in β-TI patients [26,27].

Many factors have been identified as predictors of HRQoL among NTDT patients in the current study. NTDT patients from low-income families have significantly lower scores in most HRQoL domains. Amid et al. reported that QoL is more affected in children suffering from thalassemia in low- and middle-income countries, suggesting that efforts to improve healthcare in these countries could enhance patients’ QoL [28]. The frequency of blood transfusions was negatively associated with HRQoL. This finding is similar to that reported by Mikael and Al-Allawi in Kurdistan, Iraq [22]. Age was another predictor of the QoL for NTDT patients. This correlation can be attributed to the increased frequency of complications with age and the transition to adolescence and puberty [1].

Limitations of the study

One limitation is that this study was cross-sectional. A longitudinal study is necessary to evaluate the HRQoL for each patient during the various stages and throughout the progression of the disease.

## Conclusions

Although pediatric patients with NTDT were found to have lower HRQoL scores than healthy adolescents, their QoL was superior to that of the patients with β-TM. Moreover, patients with HbH disease displayed a better HRQoL than those with β-TI patients. The frequency of blood transfusions and age were factors that had a negative association with HRQoL domains in NTDT patients. Additionally, family income was seen to negatively impact HRQoL. As such, integrating the HRQoL assessment using a standardized instrument in the care of NTDT patients can help to pinpoint the areas where patients’ functions are impaired. This can assist in making timely interventions and further improve their QoL. A longitudinal study is recommended to assess the HRQoL for each patient during the different stages and along the course of the disease.
